# Body fat ratio as a novel predictor of complications and survival after rectal cancer surgery

**DOI:** 10.3389/fnut.2024.1398807

**Published:** 2024-08-09

**Authors:** Haiyuan Zhao, Gang Liu, Yang Li, Feixiang Lu, Nianzhao Yang, Jun Zhao

**Affiliations:** Department of General Surgery, The First Affiliated Hospital of Wannan Medical College (Yijishan Hospital of Wannan Medical College), Wuhu, China

**Keywords:** body fat ratio, postoperative rectal cancer, complication, survival, nomogram model

## Abstract

**Background:**

The present study aimed to evaluate the association between body fat ratio (BFR), visceral fat area (VFA), body mass index (BMI) and visceral fat density (VFD) and assess their reliability in assessing risk of postoperative complications and survival status in patients with rectal cancer (RC).

**Materials and methods:**

The present study retrospectively included 460 patients who underwent surgical treatment for RC at the First Affiliated Hospital of Wannan Medical College (Yijishan Hospital of Wannan Medical College, Wuhu, China) between September 2018 and July 2021. BFR, VFA, BMI, and VFD were measured and basic information, clinical data, complications and survival were recorded.

**Results:**

Statistical analysis was performed to determine optimal BFR cut-off and evaluate group differences. BFR demonstrated a significant positive correlation with VFA (*R* = 0.739) and BMI (*R* = 0.783) and significant negative correlation with VFD (*R* = −0.773). The areas under the receiver operating characteristic curve of BFR, VFA, BMI, and VFD in predicting postoperative complications in RC were all >0.7 and the optimal cut-off value of BFR was 24.3. Patients in the BFR-low group had fewer postoperative complications, lower intraoperative indices, shorter hospitalization times and lower costs than those in the BFR-high group. BFR predicted complications with high diagnostic significance and was validated by multiple models. Furthermore, patients in the BFR-high group had a longer overall survival compared with patients in the BFR-low group.

**Conclusion:**

BFR was associated with BMI, VFA, and VFD. A BFR threshold of 24.3 was correlated with decreased complications and enhanced long-term survival.

## Introduction

The global incidence of colorectal cancer (CRC) is increasing annually. CRC remains the third most common malignancy worldwide. In China, the incidence of CRC ranked fourth and fifth among all types of tumor for women and men, respectively (1). In recent years, obesity has become a global public health concern. Visceral fat deposition may affect surgical visibility, resulting in prolonged operation duration and increased intraoperative bleeding (2). Furthermore, this may contribute to higher incidence of postoperative complications, impacting the postoperative survival status (3, 4). Body mass index (BMI), a commonly used indicator for assessing obesity, lacks accuracy in reflecting accumulation and distribution of body fat. BMI provides a holistic representation of overall weight but lacks ability to differentiate between muscle and fat proportion. This may result in the same BMI for individuals with substantial morphological differences, such as leaner athletes than the general population (5). Imaging-based evaluation methods, such as visceral fat area (VFA), have proven effective in prognostic evaluation for multiple types of tumors, including gastric, liver and esophageal cancer (6). Nonetheless, measurement of VFA necessitates the use of costly spiral computed tomography (CT) scanning equipment and specialized software. Moreover, subjective errors and measurement variability are present, rendering it impractical for large-scale epidemiological investigations (7, 8). Visceral fat density (VFD) is measured through volume and weight, requiring a biopsy, which is somewhat invasive. The measurement of body fat ratio (BFR) includes bioelectrical impedance analysis (BIA) and dual-energy X-ray absorptiometry (DXA). Among these, DXA provides more accurate and reliable results, but it also shares some limitations with VFA, such as radiation exposure and high equipment costs (9). In advanced cancer patients, particularly those with rectal cancer experiencing bleeding and ascites, BIA measurements are unreliable due to changes in body fluid status. By comparison, bioelectrical impedance analysis (BIA) provides a straightforward and cost-effective method for measuring body fat ratio (BFR) as it carries no radiation risk and is well-suited for large-scale implementation (10–12). Hence, there is need for additional verification and assessment to determine if BFR is precise and reliable in assessing obesity and its potential role as a risk factor for postoperative complications in rectal cancer (RC).

Furthermore, it should be assessed whether BFR has any effect on improvement of long-term survival outcomes. Hence, the present study aimed evaluate the relationship between BFR, VFA, BMI, and VFD and their reliability in assessing the risk of postoperative complications and survival status in patients with RC.

## Materials and methods

### Clinical data

Patients who underwent surgical treatment for RC between September 2018 and July 2021 at the First Affiliated Hospital of Wannan Medical College (Yijishan Hospital of Wannan Medical College, Wuhu, China) were retrospectively recruited. The inclusion criteria were as follows: (i) RC diagnosis by imaging and pathological examination without distant metastasis; (ii) no obvious contraindications to surgery and suitability for radical RC surgery; (iii) no neoadjuvant chemoradiotherapy and (iv) complete clinical data and availability of postoperative long-term outpatient clinic and telephone follow-up information. The exclusion criteria were as follows: (i) non-RC or distant metastases confirmed by imaging and pathology; (ii) poor general condition to the extent that patients could not undergo surgical treatment or the absence of radical RC surgery, excluding colonic and ileal prophylactic stomas; (iii) no abdominal CT examination; (iv) planned neoadjuvant radiochemotherapy in preoperative consultation with the physician-in-charge and (v) incomplete BFR, BMI, VFD, and VFA values. Based on these criteria, 460 patients were included in the analysis. BFR, BMI, VFD, and VFA values as well as clinicopathological data, postoperative complication status, surgery-associated indexes and postoperative recovery and long-term survival status of patients were collected. The study protocol was approved by the Ethics Committee of the First Affiliated Hospital of Wannan Medical College (Wuhu, China; Approval No. 202220).

### Research design

**Figure 1:**
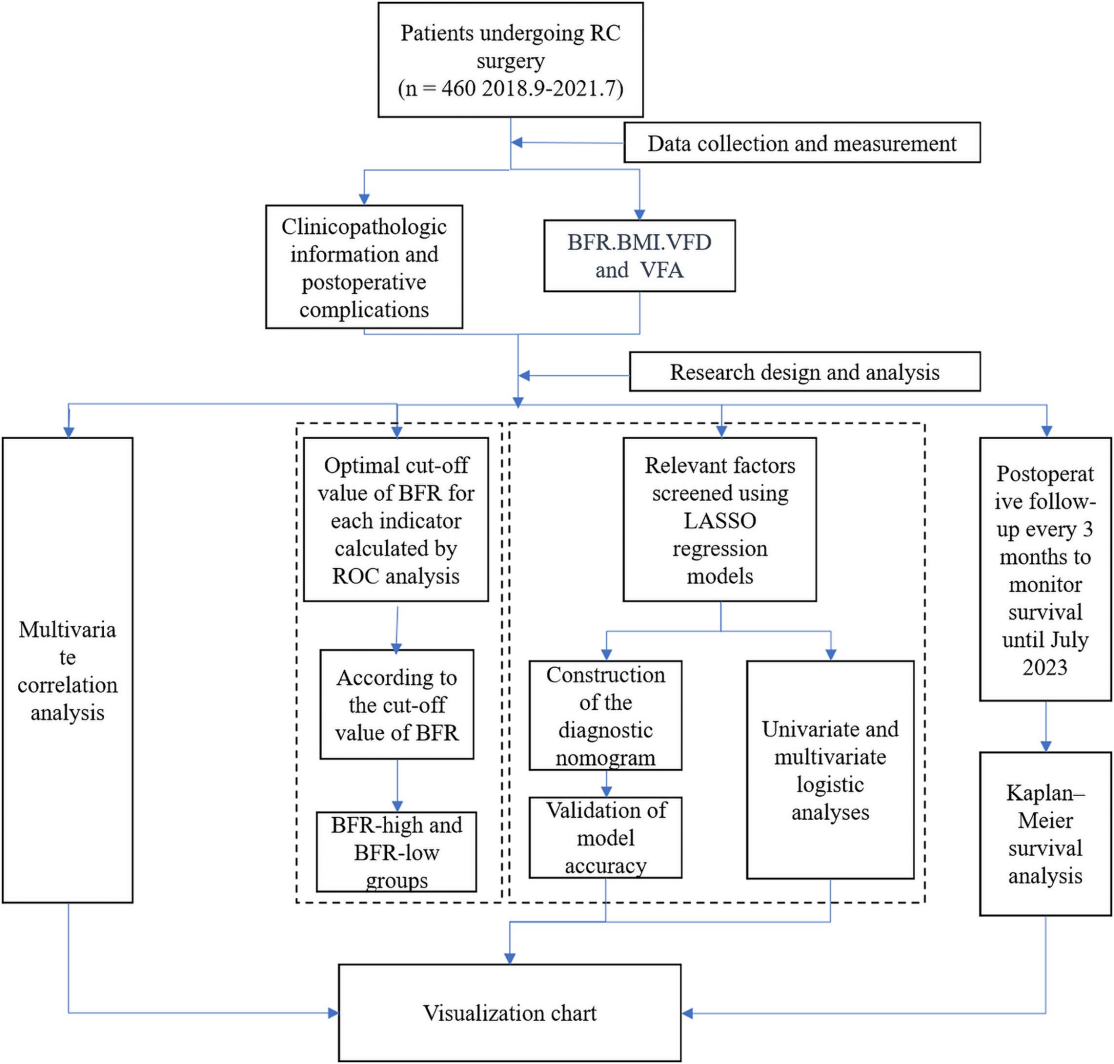
Research flowchart. RC, rectal cancer; ROC, receiver operating characteristic; BFR, body fat ratio; LASSO, least absolute shrinkage and selection operator; BMI, body mass index; VFD, visceral fat density; VFA, visceral fat area.

Patient data on BFR, BMI, VFD, and VFA values and postoperative complications were compiled using R software R software (×64 4.2.1). Correlation analysis was performed and visualization charts were generated. The accuracy of BFR, BMI, VFD, and VFA in predicting postoperative complications was assessed by receiver operating characteristic (ROC) analysis, followed by optimal cut-off value calculation for each indicator. Patients were divided into the BFR-high and-low groups according to the BFR cut-off value, and the differences in clinical data between groups were analyzed. The least absolute shrinkage and selection operator (LASSO) regression model was used to identify relevant factors. Subsequently, a diagnostic nomogram was constructed by uni-and multivariate logistic analyses and the accuracy of the model was validated. Finally, the survival status of patients was assessed using a Kaplan–Meier survival curve (Figure 1).

### Clinical measurements

The basic information of the patients was collected. Height and weight data were obtained using a SECA 213 height scale and TANITA BC-601 body composition meter to calculate BMI (kg/m^2^). BFR values were measured using a TANITA BC-601 body composition meter by BIA method (13). For VFD measurements, three postoperative pathology specimens were extracted from the omentum majus. Subsequently, volume and weight measurements were collected at three random locations (5 cm^2^) on the omentum majus using a volumetric cup as follows: VFD value = weight (g)/volume (cm^2^). The mean value of the three measurements was taken as the final VFD value. VFA was measured by abdominal spiral CT scanning and abdominal CT images at the third lumbar level were captured using Slice-O-Matic software. Fat and muscle tissues were differentiated based on CT values measured in Hounsfield Units (HU), with fat tissues having HU values between −190 and −30, and muscle tissues having HU values between −29 and 150. This differentiation allowed for the precise calculation of VFA.

### Preoperative preparation and surgical approach

All patients underwent preoperative examination, pre-perioperative evaluation and symptomatic treatment. Conventional laparoscopic surgery was performed in the lying lithotomy position under general anesthesia. A lens was placed for abdominal exploration to detect tumor location, perirectal condition, and lymph node metastasis. Conventional laparotomy was performed through a lower abdominal incision and lymph node dissection and digestive tract reconstruction were performed using a previously described method (14).

### Postoperative complications and follow-up visits

Postoperative complications observed included incisional fat liquefaction, anastomotic fistula, postoperative bleeding and bowel obstruction, anastomotic stenosis, abdominal and respiratory infection and postoperative voiding dysfunction. Survival status was routinely followed up every 3 months up to July 2023, with surgery time serving as the starting point.

### Statistical analysis

Categorical variables were expressed as counts (percentages) and analyzed using Pearson’s chi-squared test or Fisher’s exact test. Continuous variables are expressed as mean (standard deviation) or median (interquartile range) and were analyzed using a *t*-test or non-parametric test. Statistical analyses were performed using IBM SPSS Statistics 23.0 (IBM Corp.), Excel 2019 (Microsoft Corporation) and GraphPad Prism 9.3 (Dotmatics). Spearman’s correlation, ROC, Kaplan–Meier survival and univariate and multivariate logistic analyses were performed using R (version 4.2.1) with the Bioconductor package. Build a binary Logistic model using the glm function, and visualize the related model using the rms package to construct a nomogram. The log-rank test was used to analyze Kaplan–Meier survival curves. *p* < 0.05 was considered to indicate a statistically significant difference.

## Results

### Baseline characteristics of the patient population

Four hundred sixty patients had a median age of 59 years, with males comprising 36.3% of the cohort. Key baseline characteristics included a median BFR of 21.72, a median BMI of 24.37 kg/m^2^, an average VFA of 104.51 m^2^, and an average Hbg level of 100.3 g/L (Table 1).

**Table 1:** Baseline characteristics.

	Baseline characteristics (*n* = 460)
Age (years; median (IQR))	59 (44, 71)
Sex (male)	167 (36.3%)
ECOG	
0	246 (53.5%)
1	161 (35%)
2	53 (11.5%)
Smoke	271 (58.9%)
Comorbidities	
Pulmonary	38 (8.3%)
Cardiovascular	30 (6.5%)
Hypertension	76 (16.5%)
Diabetes	57 (12.4%)
Others	23 (5%)
Multiple	30 (6.5%)
BFR [median (IQR)]	21.72 (18.65, 25.26)
BMI [kg/m^2^; median (IQR)]	24.37 (22.52, 26.29)
VFA [m^2^; mean (SD)]	104.51 ± 19.16
VFD [g/cm^3^; median (IQR)]	0.78 (0.71, 0.84)
Hbg [g/L; mean (SD)]	100.3 ± 19.69
CEA [ng/mL; median (IQR)]	7.175 (2.52, 10.86)
Alb [g/L; mean (SD)]	37.14 ± 4.02

### Spearman correlation analysis of BFR, BMI, VFD, and VFA indicators

**Figure 2:**
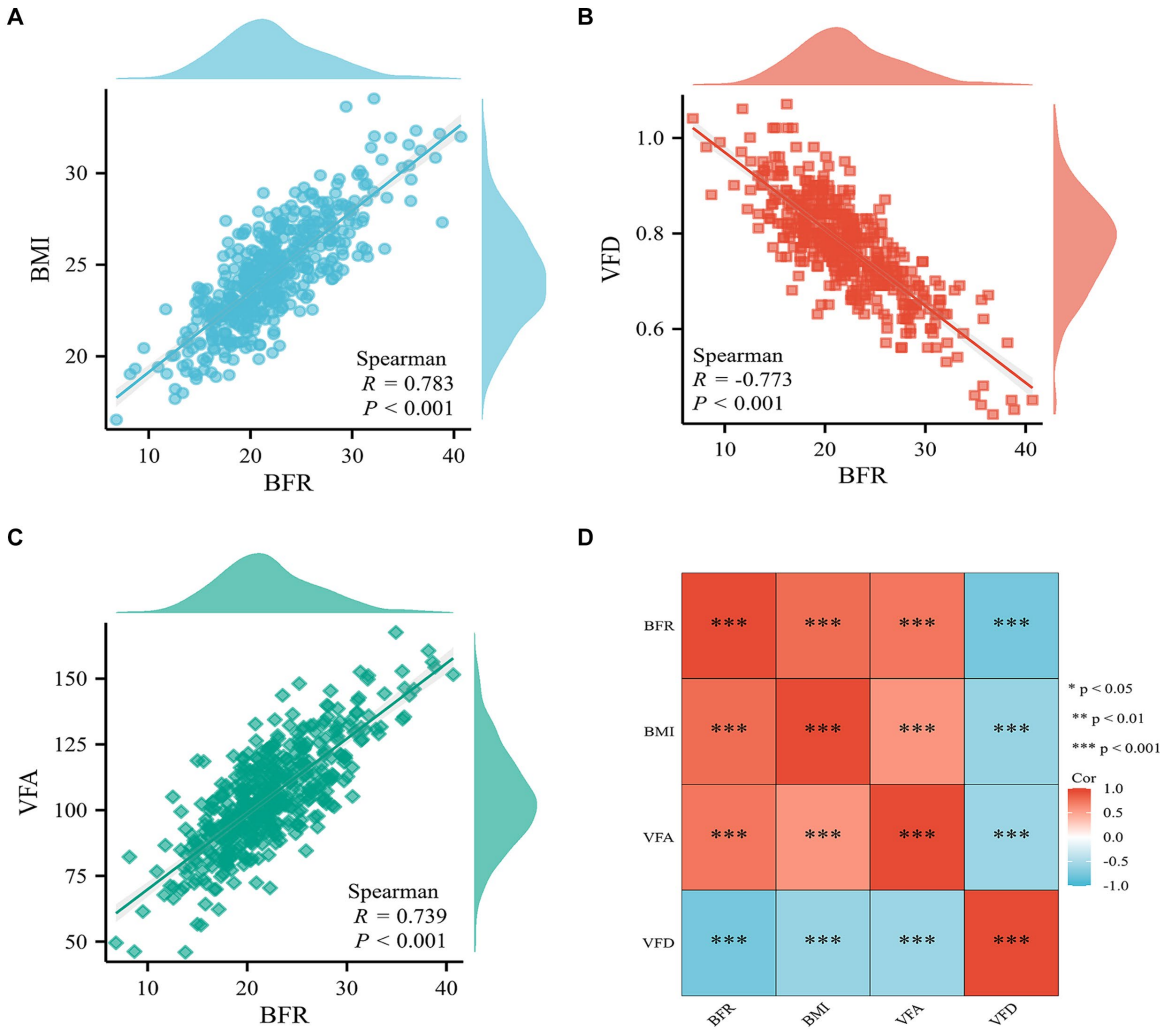
Correlation analysis of BFR, BMI, VFD, and VFA indicators. Scatter plot of correlation between BFR and **(A)** BMI, **(B)** VFD, and **(C)** VFA. **(D)** Heat map of the correlation between BFR, BMI, VFD, and VFA. BFR, body fat ratio; BMI, body mass index; VFD, visceral fat density; VFA, visceral fat area; Cor, correlation coefficient.

Significant positive correlations were demonstrated between BFR and both BMI and VFA. BFR also demonstrated a significant negative correlation with VFD (Figure 2).

### Postoperative complications

The overall incidence of postoperative complications was significantly higher in the BFR-high than that in the BFR low group. Specifically, the incidence of incisional fat liquefaction, postoperative bleeding, anastomotic fistula and chylous leakage was significantly different between the two groups. However, Clavien–Dindo grade did not exhibit a statistically significant difference between both groups (Supplementary Table S1). Furthermore, there were no significant differences in postoperative complications between the two patient groups (Table 2).

**Table 2:** Postoperative complications.

Complication (%)	BFR-low	BFR-high	*χ* ^2^	*p*-value
	*n* = 324	*n* = 136		
Anastomotic fistula	7 (2.2%)	9 (6.6%)	4.42	0.040
Postoperative bleeding	7 (2.2%)	5 (3.7%)	0.37	0.540
Bowel obstruction after abdominal surgery	9 (2.8%)	4 (2.9%)	0.00	1.000
Anastomotic stenosis	3 (0.9%)	1 (0.7%)	0.00	1.000
Abdominal infection	7 (2.2%)	9 (6.6%)	4.42	0.040
Infection of the incisional wound	10 (3.1%)	16 (11.8%)	13.53	<0.001
Pulmonary infection	3 (0.9%)	4 (2.9%)	1.43	0.230
Postoperative voiding dysfunction	8 (2.5%)	2 (1.5%)	0.10	0.750
Total incidence	54 (16.6%)	50 (36.7%)	22.11	<0.001

BFR, body fat ratio.

### Accuracy of BFR, BMI, VFD, and VFA in predicting risk of complications in patients

**Figure 3:**
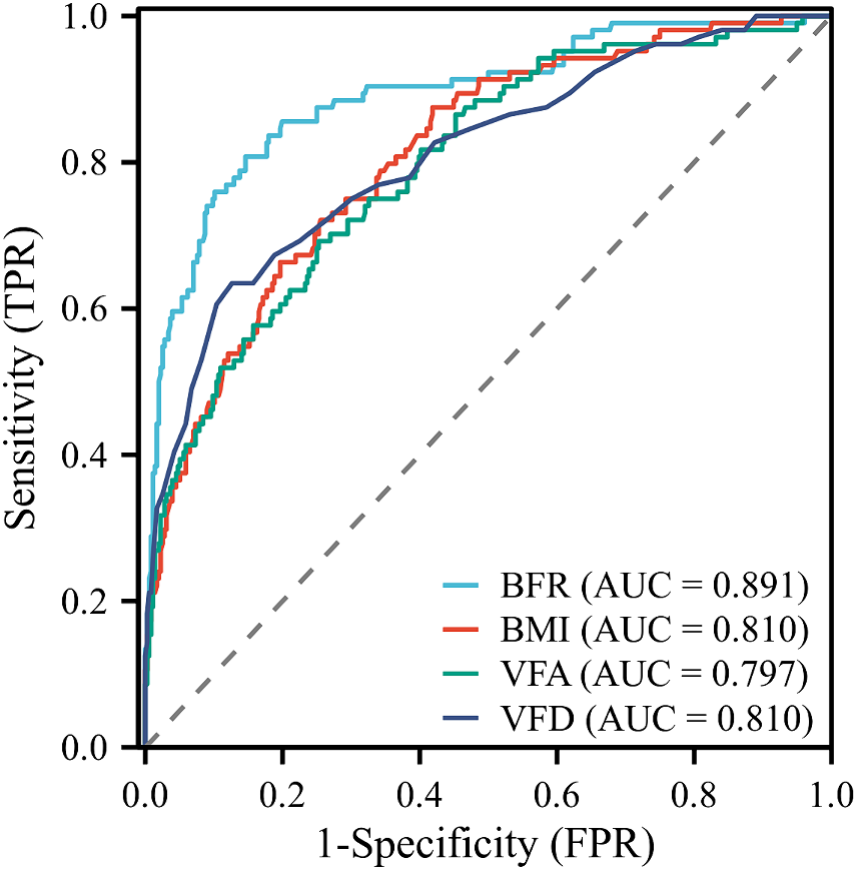
ROC curves for predicting postoperative complications in RC. AUC values for BFR, BMI, VFD, and VFA were 0.891, 0.810, 0.810, and 0.797, respectively. ROC, receiver operating characteristic; RC, rectal cancer; AUC, area under the curve; TPR, true positive rate; FPR, false positive rate.

Diagnostic ROC curves were plotted using BFR, BMI, VFD, and VFA indicators, which also predicted postoperative complications in RC. All four indicators were valuable in predicting postoperative complications of RC, with area under the ROC curve (AUC) values of 0.891, 0.810, 0.810, and 0.797, respectively (Figure 3). The optimal cutoff value determined by the Youden index based on the ROC curve. The optimal cut-off values for BFR, BMI, VFD, and VFA in predicting postoperative complications in RC were determined based on ROC curve analysis as 24.3, 25.82, 0.706, and 110.5, respectively (Supplementary Table S2).

### Clinical baseline data and pathological findings after grouping

A total of 460 patients were subjected to the final cohort analysis. The patients were categorized into BFR-low and BFR-high groups based on the optimal BFR cutoff value (24.3). No significant differences were observed between groups in terms of sex, age, smoking history, Eastern Cooperative Oncology Group scale and comorbidities (including pulmonary disease, hypertension, diabetes mellitus, other and multiple comorbidities). However, the number of patients with cardiovascular comorbidities was significantly higher in the BFR-high group compared with the BFR-low group. Furthermore, both groups were compared in terms of preoperative, intraoperative and postoperative conditions. No significant differences were observed between groups in terms of preoperative hemoglobin, carcinoembryonic antigen, and albumin.

Patients in the BFR-high group had significantly prolonged surgery time, more intraoperative blood loss, longer hospital length of stay, higher total hospital expenses and later onset of first anal exsufflation and abdominal drainage tube extubation compared with the BRF-low group. However, no significant differences were observed between groups in terms of surgical approach, ASA grading, time to first defecation and fluid intake and duration of urinary catheterization. The number of lymph nodes removed was significantly lower in the BFR-high compared with the BFR-low group. However, no significant differences were observed in tumor-node-metastases stage, degree of differentiation, neural invasion, vascular invasion, number of positive lymph nodes and distance of the tumor from the anal verge between groups (Supplementary Table S3).

### Construction of multiple diagnostic prediction models for postoperative complications in RC

**Figure 4:**
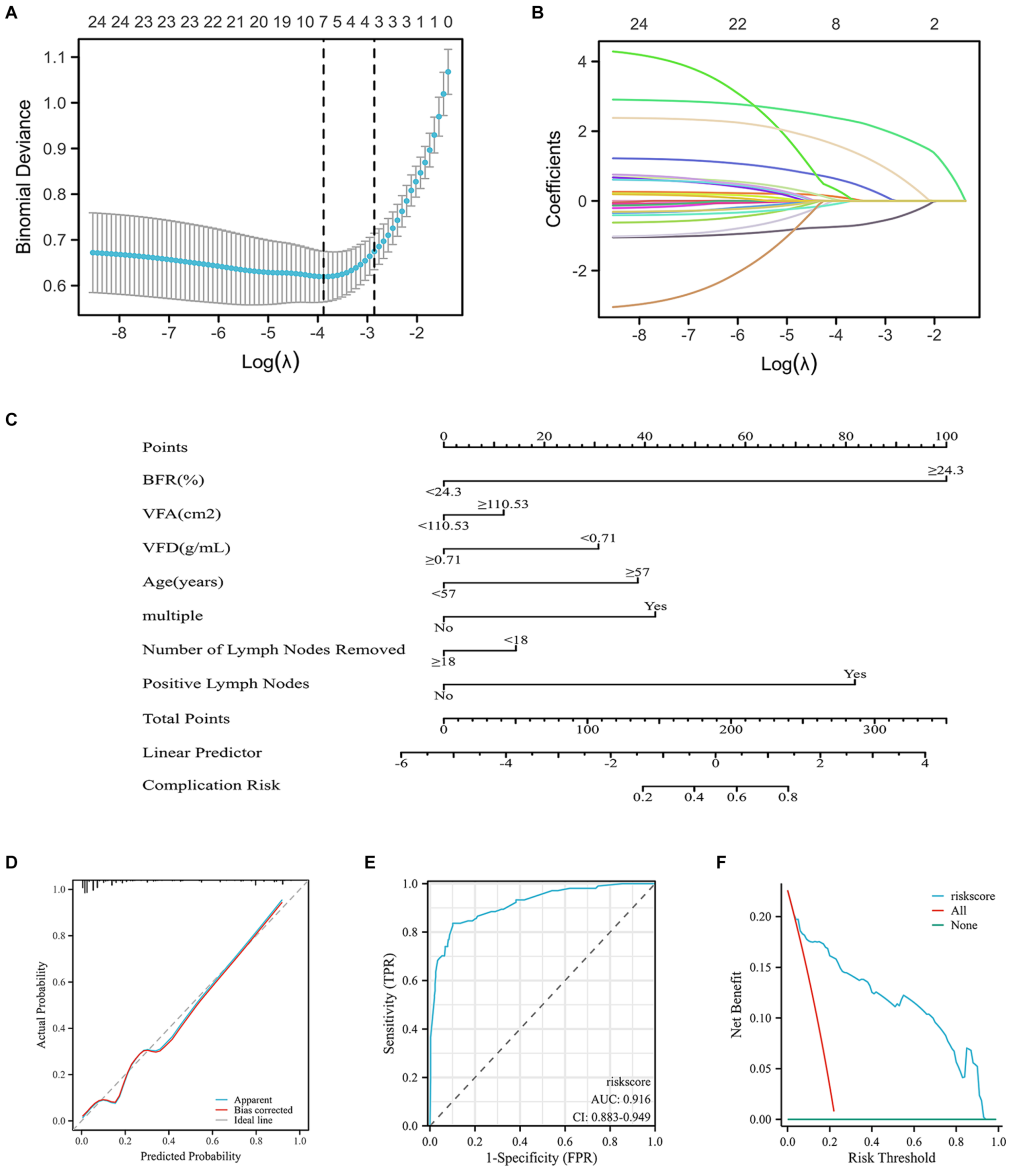
Risk factors for postoperative complications in patients with RC predicted by the nomogram model and LASSO regression analysis. **(A)** Using binomial deviation of the regularization parameter *λ* as a tuning criterion, *λ*.min was selected. Through 10-fold cross-validation, a corresponding count of non-zero coefficients was determined, amounting to 7. **(B)** Dynamic process of LASSO regression involves the filtration of coefficients for key factors. **(C)** Construction of the nomogram, encompassing all significant predictors of postoperative complications. Predictive factors included BFR, VFA, VFD, age, multiple comorbidities and number of dissected and positive lymph nodes. The cumulative sum of acceptance points for each variable is plotted on the total point axis. A line is drawn downward to the prediction axis, effectively determining the probability of the predicted outcome. **(D)** Calibration curve of the nomogram, predicting postoperative complication rates. Calibration is assessed using the Hosmer–Lemeshow goodness of fit method, demonstrating no significant disparities between predicted and observed values. This indicated a well-fitted model. **(E)** Validation of the accuracy of the nomogram by the ROC curve, demonstrating an AUC of 0.916, signifying a high level of accuracy of the model. **(F)** Clinical decision curve analysis demonstrated that the risk score curve consistently outperformed the all and none reference lines across a substantial probability threshold range. This indicated superior model performance. RC, rectal cancer; LASSO, least absolute shrinkage and selection operator; TPR, true positive rate; FPR, false positive rate; BMI, body mass index; VFD, visceral fat density; VFA, visceral fat area.

Seven variables with non-zero coefficients were screened using a LASSO regression model: BFR; VFA; VFD; age; multiple comorbidities and number of lymph nodes dissected and positive lymph nodes (Figures 4A,B and Supplementary Table S4).

A nomogram demonstrated the effect of each predictor on risk events for comorbidities, which were visualized and transformed into patient prognostic probabilities. *C*-index of 0.916 (95% confidence interval, 0.882–0.950) was recorded, suggesting high discrimination of the model (Figure 4C). The high accuracy of the nomogram was validated by diagnostic calibration graphs (Figure 4D) and ROC (Figure 4E) and decision curve analysis (DCA) curves (Figure 4F). Subsequently, these seven variables were subjected to univariate and multivariate logistic analysis. According to univariate analysis BFR, VFD, age and number of positive lymph nodes were significant risk factors. Based on multivariate analysis BFR, age and number of positive lymph nodes were significant independent risk factors for development of complications (Supplementary Table S4).

### Postoperative survival

**Figure 5:**
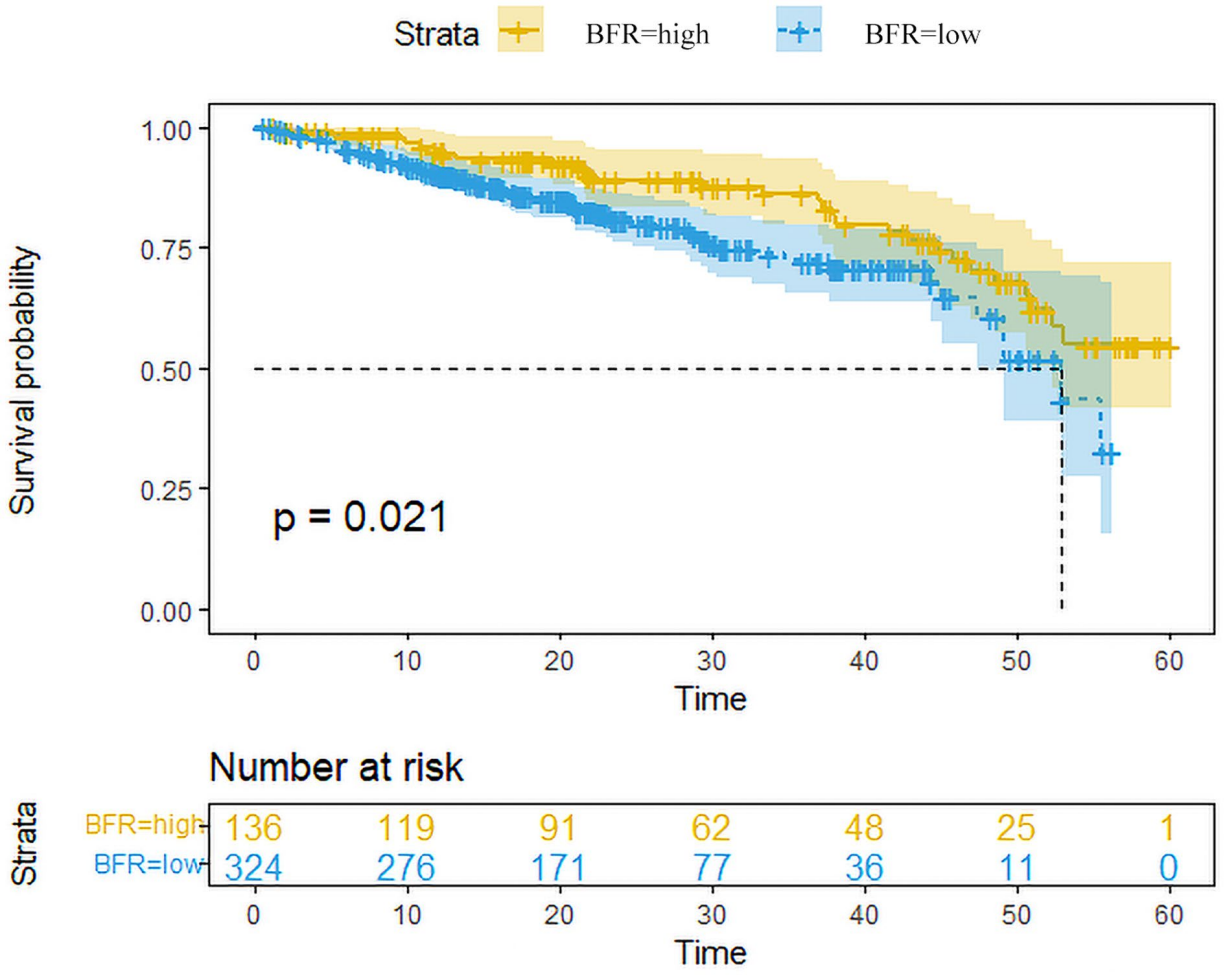
Survival curves of patients in the BFR-high and low-groups. Survival analysis conducted using the Kaplan–Meier plotter revealed that patients with high BFR (*n* = 136) exhibited a significantly lower overall survival rate compared to those with low BFR (*n* = 324) among RC patients. Excluded data points represent patients who remained alive until the end of follow-up, those who did not attend follow-up appointments and those who succumbed to other causes of mortality before the conclusion of the follow-up period. BFR, body fat ratio; RC, rectal cancer.

Data from patients with follow-up times <3 years were excluded. The 3-year survival rates of patients in the BFR-high and-low groups were 86.2 and 72.4%, respectively. Overall, the survival rate in the BFR-high group was significantly higher compared with the BFR-low group (Figure 5).

## Discussion

Obesity is a notable risk factor for numerous chronic diseases, with a particular emphasis on its association with digestive system tumors, notably RC, which has both a high incidence and poor prognosis (15). Therefore it is key to maintain vigilance against obesity to mitigate this risk (16). Obesity in patients with RC increases the risk of numerous metabolic disorders, including hypertension, cardiovascular and cerebrovascular diseases and diabetes. Furthermore, obesity compounds risk factors associated with RC (17, 18). Therefore, identification of potential prognostic markers and novel therapeutic targets for obese patients with RC is necessary (19). Despite advancements in treatment, postoperative complications and tumor recurrence affect patient survival and prognosis (20). In particular, complications may lead to difficulties in subsequent treatment, worsening prospects of patients survival (21). Therefore, improving surgical outcome and prognosis of patients with RC has become a topic of research.

Our comparative analysis revealed that BIA-based BFR is a cost-effective, practical alternative to CT-based VFA for assessing obesity and predicting postoperative complications in RC patients. BFR, despite being less precise, shows a strong correlation with VFA and can be easily implemented in clinical settings. These findings support the use of BFR as a reliable proxy for VFA in clinical practice, especially where CT scans are impractical. Future multi-center studies are needed to validate BFR’s impact on long-term survival outcomes.

Two indicators of fat distribution, VFA and BMI, have received attention and are associated with the incidence and prognosis of RC (22). However, these metrics have some limitations (23). VFA used X-rays as a source of radiation to differentiate between fat, non-fat and bone mineral mass by measuring attenuation of X-rays in different tissues of the body (24). Due to the high cost of the equipment required for VFA assessment and the necessity for specialized software systems, VFA incurs significant expenses (25). Furthermore, in this patient population, CT scans are considered the gold standard for body composition assessment, providing high accuracy and reliability (9). Consequently, although VFA assessment may be expensive, it yields accurate results. While BMI is a commonly used and straightforward measure of body mass and is positively associated with BFR, BMI cannot distinguish between fat and muscle mass and may misclassify individuals with greater muscle mass as obese. Therefore, these techniques may not accurately reflect patient body fat and impact on RC treatment.

Methods for measuring BFR include DXA and BIA. BIA results can be affected by ascites and bleeding in advanced cancer patients, whereas DXA provides more accurate results. However, this study excluded advanced cancer patients, including those with malignant ascites, hypoproteinemic ascites, and anemia caused by bleeding, to ensure the reliability of the measurements. BFR more accurately reflects the actual degree of obesity in an individual compared with BMI (15). BFR also accurately characterizes adipose tissue distribution and abnormal proportions (23). Moreover, BFR is associated with metabolic disorder and is more sensitive to changes in the intra-abdominal environment (3). To the best of our knowledge, however, studies on BFR in preoperative assessment of body mass and its effect on RC surgery are scarce. It is hypothesized that the present study is the first to propose the concept of VFD. Some large and fragile fat particles are observed in obese patients. This phenomenon increases risk bleeding and entry at inappropriate anatomical levels, thus increasing the risk of complications (26). Therefore, in the present study, the correlation between BFR, VFA, BMI, and VFD indexes was assessed and validated. Furthermore, the relationship between BFR values and postoperative complications and prognosis of RC was explored.

The present study demonstrated that BFR exhibited a significant positive correlation with VFA and BMI and a significant negative correlation with VFD. These metrics accurately reflect the degree of obesity in patients and predict postoperative complications in RC. This is consistent with previous findings that VFA and BMI are associated with intraoperative and postoperative complications and poor prognosis in patients with RC, further validating the importance of BFR and VFD (27, 28). However, the optimal diagnostic cut-off points for these metrics are different. Therefore, further optimization of selecting metrics for predicting complications at specific levels of obesity is needed. To minimize statistical errors, the optimal cut-off values obtained should be used to classify patients into BFR-high and BFR-low groups, rather than relying solely on standardized or median values of BFR. To mitigate potential errors introduced by grouping, the present study used cut-off values of BFR to delineate high and low groups. Given the variation in normal ranges of BFR across different sexes and age groups, categorizing BFR into high, normal and low groups, or tripartitioning from high to low values, would result in increased rates of true and false positives.

Multivariate logistic analysis identified BFR, age and number of positive lymph nodes as significant risk factors for postoperative complications. The nomogram visualized the impact of each predictor on risk of complications and translated it into patient prognostic probabilities. According to the nomogram, BFR value had a relatively large scale on the graph, indicating a role of BFR in determining the risk of complication. Furthermore, the model exhibited high accuracy and reliability with a corresponding *C*-index value of 0.916 (95% CI, 0.882–0.950). Seven variables were identified among those with non-zero coefficients in the LASSO regression model, including BFR, VFA, VFD, age, multiple comorbidities and number of dissected and positive lymph nodes. These variables exerted a significant impact on predicting postoperative complications in RC. These findings suggested a potential application and clinical significance of the model in predicting the risk of complications.

Clinical data demonstrated that patients in the BFR-high group had prolonged operation duration, more intraoperative bleeding and higher incidence of intraoperative complications during radical RC surgery compared with those in the BFR-low group (29). Surgeons often encounter technical limitations during RC surgery in obese patients, including poor visualization and operative difficulties, especially in men with low RC. These unfavorable factors increase difficulty of surgical dissection, prolong the time of radical RC surgery and increase risk of bleeding and complications. In addition, lymph node metastasis is a key factor affecting prognosis of patients with RC. In radical RC surgery, lymph node dissection is crucial for efficacy, serving as a key index for evaluating RC surgery. Patients in the BFR-high group may have had a lower number of lymph nodes dissected. However, a previous study reported that BMI does not influence intraoperative lymph node dissection (30). High BFR may increase the difficulty of intraoperative lymph node dissection and decrease the effectiveness of dissection, thereby affecting the efficacy of RC surgery. In summary, BFR exerts a key effect on the outcome of RC surgery. High BFR values increase the difficulty of surgery and the risk of intraoperative complications (31). Moreover, higher BFR may decrease the effect of peristomal lymph node dissection, thereby impacting the efficacy of RC surgery (17). Therefore, for obese patients, especially those with high BFR, it is suggested that enhanced operating skills are required in RC surgery to improve outcomes.

VFA and BMI are used to decrease the risk of surgical complications and improve survival in patients with RC (32). Patients in the BFR-high group had an increased risk of RC surgical complications, which adversely affected postoperative recovery. These patients exhibited significantly prolonged hospitalization time, increased operation duration and intraoperative bleeding, prolonged anal ventilation, delayed dietary recovery, increased hospitalization costs and decreased number of dissected lymph nodes. To decrease the risk of these complications, especially in patients with RC with comorbid metabolic syndrome, preoperative measures should be taken to decrease BFR. Korhonen et al. (25) reported that adiponectin, an adipokine with protective effects, may lead to surgical complications, including surgical site infection and pneumonia. Hypertension and dyslipidemia are associated with impaired microvascular circulation, which may lead to poor tissue healing and increased risk of wound complications and anastomotic leakage. Although each factor exerts a small effect on postoperative outcomes, the risk of postoperative complications is significantly increased when multiple factors are present. Future studies should investigate the association between BFR and postoperative inflammatory markers, thus validating the role of BFR in RC surgery.

Low BFR may lead to poor prognosis in patients with RC. This may be attributed to the nutritional status of the patient. Good nutritional status helps to combat unfavorable risks and promotes long-term survival. Hence, malnourished patients tend to have poorer prognosis. Therefore caution is needed when considering interventions to decrease BFR. Visceral fat may regulate tumor cell behavior such as proliferation, adhesion and invasion by influencing adipocytokines. Low adiponectin levels are associated with development of malignant tumors and poor prognosis, as adiponectin serves an anticancer role by inhibiting tumor cell proliferation and promoting apoptosis (33).

Furthermore, whether high BFR effects patient prognosis solely via postoperative complications remains controversial, which is a limitation of this study. Moreover, the sample size of the present single-center study was small, thus warranting future multicenter, large-sample studies. In patients with RC, BFR was positively correlated with VFA and BMI but negatively correlated with VFD. However, the present study did not explore the correlation between these four factors in healthy individuals, which is another limitation. To enhance the completeness and accuracy of the present results, further experiments should be performed to assess the correlation among these four factors in the healthy population. Moreover, the present study on the relationship between BFR and tumors remained at the level of clinical research. The underlying mechanism, especially its association with indicators reflecting obesity *in vivo*, such as lipids, serum leptin, adiponectin and their receptors, has not yet been fully evaluated. Therefore, these aspects need to be studied to further the understanding of BFR in prognosis of patients with RC.

## Conclusion

In summary, accurate assessment of BFR, VFA, VFD, and BMI can clarify the degree of obesity and fat distribution in patients with RC. The selection of appropriate surgical plans and postoperative management strategies provides novel ideas and reliable support for development of personalized treatment strategies, thereby reducing complications and extending their overall survival.

## Data Availability

The original contributions presented in the study are included in the article/Supplementary material, further inquiries can be directed to the corresponding author.
