# Supplementary material for: Body fat ratio as a novel predictor of complications and survival after rectal cancer surgery
Source: Front Nutr. 2024 Aug 9;11:1398807. doi: 10.3389/fnut.2024.1398807 (PMC11341451; doi:10.3389/fnut.2024.1398807)
Supplement: Supplementary file 1 [file Table_1.DOCX]

**Supplementary data**

**The supplementary tables are as follows.**

**Supplementary Table 1: AUC and cut-off values for diagnostic ROC curves**

| Predictor variables | Predictive outcomes | AUC | CI | Cut-off value |
| --- | --- | --- | --- | --- |
| BMI | Positive | 0.81 | 0.764–0.857 | 25.82 |
| VFA | Positive | 0.797 | 0.748–0.846 | 110.53 |
| VFD | Negative | 0.81 | 0.760–0.860 | 0.706 |
| BFR | Positive | 0.891 | 0.852–0.930 | 24.3 |

Abbreviations: AUC, area under the curve; ROC, receiver operating characteristic; CI, confidence interval; BMI, body mass index; VFA, visceral fat area; VFD, visceral fat density; BFR, body fat ratio.

**Supplementary Table 2: Clinical data for both groups**

| **Characteristics** | **Low** | **High** | **Statistic** | ***P*-value** |
| --- | --- | --- | --- | --- |
|  | **n = 324** | **n = 136** |  |  |
| Sex |  |  | 0.31 | 0.58 |
| Female | 209 (64.5%) | 84 (61.8%) |  |  |
| Male | 115 (35.5%) | 52 (38.2%) |  |  |
| Age, years | 59 (45, 71) | 57 (42, 71) |  | 0.59 |
| ECOG |  |  | 1.14 | 0.57 |
| 0 | 175 (54%) | 71 (52.2%) |  |  |
| 1 | 115 (35.5%) | 46 (33.8%) |  |  |
| 2 | 34 (10.5%) | 19 (14%) |  |  |
| Smoke | 186 (57.4%) | 85 (62.5%) | 1.03 | 0.31 |
| Comorbidities |  |  |  |  |
| Pulmonary | 25 (7.7%) | 13 (9.6%) | 0.43 | 0.51 |
| Cardiovascular | 12 (3.7%) | 18 (13.2%) | 14.28 | 0.00 |
| Hypertension | 48 (14.8%) | 28 (20.6%) | 2.32 | 0.13 |
| Diabetes | 40 (12.3%) | 17 (12.5%) | 0.00 | 0.96 |
| Others | 15 (4.6%) | 8 (5.9%) | 0.32 | 0.57 |
| Multiple | 25 (7.7%) | 5 (3.7%) | 2.56 | 0.11 |
| Hbg，g/L | 99.91 ± 19.12 | 101.25 ± 21.04 | -0.67 | 0.51 |
| CEA，ng/ml | 7.27 (2.73, 11.11) | 7.12 (1.56, 10.12) | | 0.45 |
| Alb，g/L | 37.27 ± 4.03 | 36.84 ± 3.99 | 1.05 | 0.30 |
| BMI, kg/m^2^ | 23.25 (21.94, 24.76) | 27.04 (25.6, 28.48) | | <0.001 |
| VFA，m^2^ | 97.33 ± 15.39 | 121.62 ± 16.24 | -15.20 | <0.001 |
| VFD， g/cm^3^ | 0.81 (0.77, 0.86) | 0.69 (0.63, 0.72) | | <0.001 |
| Distance from the anal margin |  |  | 0.59 | 0.44 |
| <7 cm | 207 (63.9%) | 92 (67.6%) |  |  |
| ≥7 cm | 117 (36.1%) | 44 (32.4%) |  |  |
| TNM stage |  |  | 0.64 | 0.42 |
| Ⅰ–Ⅱ | 245 (75.6%) | 98 (72.1%) |  |  |
| Ⅲ | 79 (24.4%) | 38 (27.9%) |  |  |
| Differentiation grade |  |  | 0.09 | 0.76 |
| Medial-high | 285 (88%) | 121 (89%) |  |  |
| Low | 39 (12%) | 15 (11%) |  |  |
| Number of lymph nodes removed | 19 (18, 20) | 16 (15, 18) |  | 0.00 |
| Positive lymph nodes | 206 (63.6%) | 86 (63.2%) | 0.00 | 0.94 |
| Neural invasion | 54 (16.7%) | 30 (22.1%) | 1.87 | 0.17 |
| Vascular invasion | 46 (14.2%) | 11 (8.1%) | 3.29 | 0.07 |
| Operative approach |  |  | 0.74 | 0.39 |
| Laparoscopic surgery | 276 (85.2%) | 120 (88.2%) |  |  |
| Laparotomy | 48 (14.8%) | 16 (11.8%) |  |  |
| ASA grade, |  |  | 0.28 | 0.87 |
| 1 | 121 (37.3%) | 52 (38.2%) |  |  |
| 2 | 107 (33%) | 47 (34.6%) |  |  |
| 3 | 96 (29.6%) | 37 (27.2%) |  |  |
| Surgery time,min | 140.45 ± 38.59 | 183.9 ± 27.08 | -13.75 | <0.001 |
| Intraoperative blood loss, mL | 112.25 (82.18, 142.98) | 151.85 (110.57, 191.8) | | <0.001 |
| Anal exhaustion time, days | 2.1 (1.5, 2.9) | 2.7 (1.9, 3.3) |  | <0.001 |
| Defecation time, days | 6.75 ± 1.88 | 6.93 ± 2.21 | -0.84 | 0.4 |
| Time of first liquid intake, days | 1.99 ± 0.71 | 1.87 ± 0.71 | 1.71 | 0.09 |
| Duration of urinary catheterization, days | 3.35 ± 1.47 | 3.53 ± 1.45 | -1.20 | 0.23 |
| Hospital length of stay, days | 10.12± 3.41 | 11.56 ± 1.93 | -5.73 | <0.001 |
| Total hospital expenses, RMB | 3.9 (3.1, 4.7) | 5.95 (5.6, 6.6) |  | <0.001 |
| Drainage tube removal time, days | 6 (5.3, 6.7) | 7.15 (6.1, 8.8) |  | <0.001 |

Abbreviations: ECOG, Eastern Cooperative Oncology Group; Hbg, hemoglobin; CEA, carcinoembryonic antigen; Alb, albumin; BMI, body mass index; VFA, visceral fat area; VFD, visceral fat density; TNM, tumor-node-metastases; ASA, American Society of Anesthesiologists.

**Supplementary Table 3: Diagnostic LASSO coefficient screening table**

| Variable | Lambda.min | Lambda.1se |
| --- | --- | --- |
| (Intercept) | -3.04 | -2.21 |
| BFR | 2.35 | 1.95 |
| BMI | 0 | 0 |
| VFA | 0.1 | 0 |
| VFD | -0.73 | -0.51 |
| Sex | 0 | 0 |
| ECOG | 0 | 0 |
| Age | 0.59 | 0.03 |
| Smoke | 0 | 0 |
| Pulmonary | 0 | 0 |
| Cardiovascular | 0 | 0 |
| Hypertension | 0 | 0 |
| Diabetes | 0 | 0 |
| Others | 0 | 0 |
| Multiple | 0.23 | 0 |
| Anal margin | 0 | 0 |
| TNM stage | 0 | 0 |
| Differentiation grade | 0 | 0 |
| Number of lymph nodes removed | -0.06 | 0 |
| Maximum tumor diameter | 0 | 0 |
| Positive lymph nodes | 1.54 | 0.83 |
| Neural invasion | 0 | 0 |
| Vascular invasion | 0 | 0 |
| Prophylactic fistula | 0 | 0 |
| Operative approach | 0 | 0 |
| ASA grade | 0 | 0 |

Abbreviations: LASSO, least absolute shrinkage and selection operator; BMI, body mass index; VFA, visceral fat area; VFD, visceral fat density; ECOG, Eastern Cooperative Oncology Group; TNM, tumor-node-metastases; ASA, American Society of Anesthesiologists.

**Supplementary Table 4: Univariate and multivariate logistic analyses of postoperative complications of RC**

| Characteristics | Univariate analysis | *P*-value | Multivariate analysis | *P*-value |
| --- | --- | --- | --- | --- |
| BFR | 24.554 (13.893 - 43.395) | <0.001 | 13.873 (6.158 - 31.254) | <0.001 |
| VFA | 0.150 (0.093 - 0.243) | <0.001 | 0.718 (0.337 - 1.527) | 0.389 |
| VFD | 12.004 (7.230 - 19.929) | <0.001 | 2.328 (1.081 - 5.014) | 0.031 |
| Age | 2.582 (1.651 - 4.037) | <0.001 | 3.040 (1.582 - 5.839) | <0.001 |
| Multiple | 1.265 (0.546 - 2.932) | 0.583 |  |  |
| Number of lymph  nodes removed | 3.473 (2.127 - 5.670) | <0.001 | 1.509 (0.751 - 3.032) | 0.248 |
| Positive lymph nodes | 0.131 (0.073 - 0.236) | <0.001 | 0.109 (0.052 - 0.227) | <0.001 |

Abbreviations: RC, rectal cancer; BFR, body fat ratio; VFA, visceral fat area; VFD, visceral fat density.
